# Supplementary material for: Splice-Junction-Based Mapping of Alternative Isoforms in the Human Proteome
Source: Cell Rep. Author manuscript; Available in PMC 2020 Jan 15. (PMC6961840; doi:10.1016/j.celrep.2019.11.026)
Supplement: 2 [file NIHMS1546469-supplement-2.zip › DF1/PXD010154/Thyroid/Thyroid_44_SLC25A11_AGPLVPSLAGGIDGKPRTSPK.pdf]

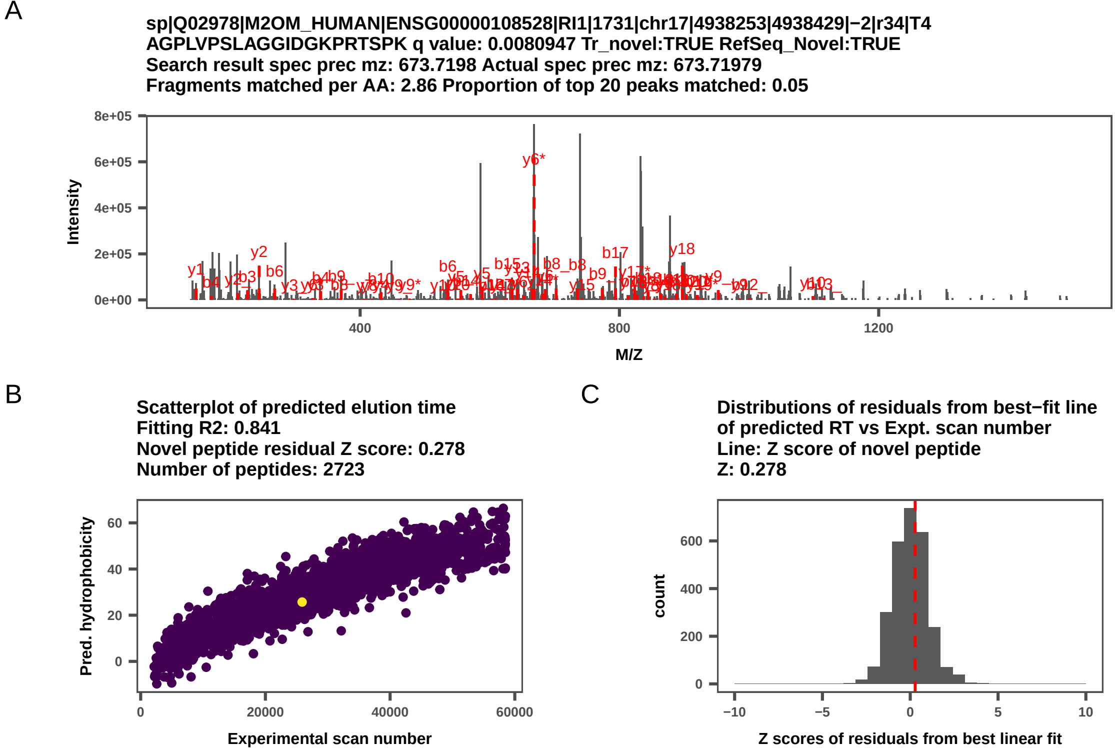
A
sp|Q02978|M2OM_HUMAN|ENSG00000108528|RI1|1731|chr17|4938253|4938429|−2|r34|T4
AGPLVPSLAGGIDGKPRTSPK q value: 0.0080947 Tr_novel:TRUE RefSeq_Novel:TRUE
Search result spec prec mz: 673.7198 Actual spec prec mz: 673.71979
Fragments matched per AA: 2.86 Proportion of top 20 peaks matched: 0.05
Intensity
8e+05
6e+05
4e+05
2e+05
0e+00
y6*
y2
y1
b6
b17
y18
M/Z
400
800
1200
B
Scatterplot of predicted elution time
Fitting R2: 0.841
Novel peptide residual Z score: 0.278
Number of peptides: 2723
Pred. hydrophobicity
60
40
20
0
0
20000
40000
60000
Experimental scan number
C
Distributions of residuals from best−fit line
of predicted RT vs Expt. scan number
Line: Z score of novel peptide
Z: 0.278
count
600
400
200
0
−10
−5
0
5
10
Z scores of residuals from best linear fit
